# Utilizing Raman spectroscopy for urinalysis to diagnose acute kidney injury stages in cardiac surgery patients

**DOI:** 10.1080/0886022X.2024.2375741

**Published:** 2024-07-12

**Authors:** Mukta Sharma, Chia-Lung Tsai, Ying-Chang Li, Cheng-Chia Lee, Yu-Li Hsieh, Chih-Hsiang Chang, Shao-Wei Chen, Liann-Be Chang

**Affiliations:** a Graduate Institute, Prospective Technology of Electrical Engineering and Computer Science, National Chin-Yi University of Technology, Taiwan; b Department of Electronic Engineering, Chang Gung University, Taoyuan, Taiwan; c Department of Otolaryngology-Head and Neck Surgery, Chang Gung Memorial Hospital, Taoyuan, Taiwan; d Department of Electronic Engineering, Ming Chi University of Technology, New Taipei City, Taiwan; e Department of Nephrology, Kidney Research Center, Chang Gung Memorial Hospital, Taoyuan, Taiwan; f Graduate Institute of Clinical Medical Sciences, College of Medicine, Chang Gung University, Taoyuan, Taiwan; g Department of Electrical and Electronic Engineering, Chung Cheng Institute of Technology, National Defense University, Taoyuan, Taiwan; h Department of Cardiothoracic and Vascular Surgery, Chang Gung Memorial Hospital, Taoyuan, Taiwan; i Green Technology Research Center, Chang Gung University, Taoyuan, Taiwan

**Keywords:** Acute kidney injury, cardiac surgery, Raman spectroscopy, partial least squares, support vector machine, urine

## Abstract

**Background:**

The successful treatment and improvement of acute kidney injury (AKI) depend on early-stage diagnosis. However, no study has differentiated between the three stages of AKI and non-AKI patients following heart surgery. This study will fill this gap in the literature and help to improve kidney disease management in the future.

**Methods:**

In this study, we applied Raman spectroscopy (RS) to uncover unique urine biomarkers distinguishing heart surgery patients with and without AKI. Given the amplified risk of renal complications post-cardiac surgery, this approach is of paramount importance. Further, we employed the partial least squares-support vector machine (PLS-SVM) model to distinguish between all three stages of AKI and non-AKI patients.

**Results:**

We noted significant metabolic disparities among the groups. Each AKI stage presented a distinct metabolic profile: stage 1 had elevated uric acid and reduced creatinine levels; stage 2 demonstrated increased tryptophan and nitrogenous compounds with diminished uric acid; stage 3 displayed the highest neopterin and the lowest creatinine levels. We utilized the PLS-SVM model for discriminant analysis, achieving over 90% identification rate in distinguishing AKI patients, encompassing all stages, from non-AKI subjects.

**Conclusions:**

This study characterizes the incidence and risk factors for AKI after cardiac surgery. The unique spectral information garnered from this study can also pave the way for developing an *in vivo* RS method to detect and monitor AKI effectively.

## 1. Introduction

Acute kidney injury (AKI) after cardiac surgery is a significant health issue related to increased morbidity, mortality, and healthcare costs. It affects up to 40% of patients following cardiac surgery, with 1% of cases necessitating dialysis treatment [1]. It can lead to longer hospital stays, higher risk of complications, and poorer patient outcomes. Preventive strategies, early detection, and optimized treatment approaches are crucial for minimizing the impact of AKI after cardiac surgery. AKI is a noninfectious and potentially treatable yet severe clinical condition that impacts approximately 5–13% of the global population [2]. Minimizing the effects of AKI can be achieved through prompt identification and the introduction of straightforward preventative measures to prevent disease progression. Regrettably, AKI diagnosis is often subject to delays or misdiagnosis [3]. Current clinical parameters used to estimate renal function, including serum creatinine and urine volume, only become measurable after a considerable decline in kidney function has occurred and persisted for a certain period [4]. The successful treatment and improvement of AKI completely depend on early stage diagnosis and prompt intervention, emphasizing the importance of timely detection and appropriate action in managing the condition [5]. The diagnosis of AKI depends on blood tests measuring creatinine and blood urea levels, as well as assessment of glomerular filtration rate, urine output, urinary markers of kidney injury, and occasionally kidney biopsies [6]. It is important to recognize that no single biomarker is near-perfect for AKI detection due to its multiple etiology. Each identified biomarker has its advantages and disadvantages. However, the concurrent detection of multiple biomarkers can significantly improve early and accurate diagnosis of AKI. Up to now, various techniques have been reported for detecting protein-like biomarkers. Late diagnosis of AKI is linked to disease progression, leading to costly treatments, delayed recovery, and a higher mortality rate [7]. This underscores the need for a simple, rapid, and nondestructive diagnostic method for AKI detection. In the presence of kidney disease, there are alterations in the chemical composition and structure of compounds found in urine [8]. Significant studies have been carried out on urine biomarkers for the identification of chronic renal disease [9]. Urine is especially significant because it enables a noninvasive and painless sample collection method, and it contains over 3000 metabolites or metabolic species that can be utilized for diagnostic purposes [10]. The most prevalent organic chemicals in urine consist of urea, creatinine, uric acid, hippuric acid, and citric acid.

Raman spectroscopy (RS) is a prominent label-free technique used for obtaining fingerprint-like information on molecules and conducting biological analyses, due to its outstanding sensitivity, selectivity, and biocompatibility. Analyzing urine with RS provides multiple benefits compared to traditional chemical methods, including no requirement for dilution and reagents, reduced evaluation times, more extensive information, and the use of smaller sample volumes [11]. Raman spectroscopy, when combined with robust machine learning algorithms, is frequently employed for biomolecular detection [12]. In nearly every disease, initial changes take place at the molecular level [13–18]. Such techniques assist in tracking subtle spectral variations linked to specific diseases. Moreover, they offer rapid and nondestructive analyses without the need for sample preparation, making them ideal for screening tests. Numerous instances in the literature demonstrate the successful use of biofluid-based RS for screening various types of cancer [19–26]. In a previous study [18], it showed that RS, combined with partial least squares-linear discriminant analysis (PLS-LDA), can successfully distinguish AKI patients from non-AKI patients using urine samples. The present study encompassed 200 consecutive patients who underwent cardiac surgery. The objective of this study to analyze the Raman spectra of urine samples in order to differentiate between three stages of AKI and non-AKI patients following heart surgery, with the goal of improving kidney disease management in the future. We have developed a quantitative model utilizing partial least squares-support vector machine (PLS-SVM) that can be applied to urine Raman spectra for assessing the concentrations of key metabolites in the body. This method has the potential to enhance AKI diagnosis and detect stage bias, ultimately contributing to early detection of AKI.

## 2. Methods and materials

### 2.1. Patients and samples

This study was granted approval by the Chang Gung Memorial Hospital’s Institutional Review Board (IRB) under the IRB numbers 103-1993B, 202001691B0, and 202301071B0. The research took place between June 2014 and July 2017 in the cardiac surgery intensive care unit (ICU) of a tertiary care referral center in Taiwan. Clinical data and pathological reports were gathered from the Department of Nephrology at Chang Gung Memorial Hospital. A total of 200 urine samples were utilized in the study. The average baseline serum creatinine level was 1.1 ± 0.7 mg/dL. Participants were admitted to the ICU immediately following cardiac surgery and provided written informed consent for urine sample collection. Exclusion criteria included patients on dialysis, those under 20 years of age, those with an estimated glomerular filtration rate below 30 mL/min/1.73 m^2^, previous organ transplant recipients, or those experiencing anuria immediately post-surgery. Fresh urine samples were collected in sterile, non-heparinized tubes within the first 4 h after surgery. Samples were then centrifuged at 5000 × *g* for 30 min at 4 °C to eliminate cells and debris. The most frequent cardiac surgery performed was coronary artery bypass graft (CABG), followed by heart valve surgery, aorta surgery, and CABG combined with valve surgery. Within the study cohort, 171 patients (85.5%) underwent on-pump cardiac surgery, while 29 patients (14.5%) underwent off-pump cardiac surgery. For those patients who underwent on-pump cardiac surgery, a uniform priming solution was administered. Within the study cohort, 29 patients (14.5%) underwent emergency surgery. Out of 200 patients, postoperative AKI was observed in 80. Of these, 42 patients were diagnosed with AKI stage 1, 19 with AKI stage 2, and 19 with AKI stage 3. All consecutive eligible patient demographics can be found in Table 1. The mean EUROSCORE II of the patients included in the study is 4.6 ± 5.6. The pre-operative glomerular filtration rate was 80.7 ± 27.9 mL/min/1.73 m^2^. Among the study patients, 122 patients (61.0%) have hypertension, 77 patients (38.5%) have diabetes, and 16 patients (8.0%) have chronic obstructive pulmonary disease (COPD). Among our study patients, 78 patients (39%) used diuretics between 24 h before cardiac surgery and within 6 h after surgery. The main goal was to assess the ability of RS to differentiate AKI from non-AKI cases, focusing on the development of AKI within seven days post-cardiac surgery as the primary outcome. AKI development was determined using the serum creatinine criteria outlined in the Kidney Disease Improving Global Outcomes (KDIGO) Clinical Practice Guidelines for AKI. Urine samples were placed in centrifuge tubes and stored at −70 °C to preserve their morphology until use. Before measurements, the samples were thawed to room temperature. A 30 µL sample of urine was collected for measurement and placed on an aluminum foil-based substrate. Multiple spectra were obtained at different locations within the sample. Overall, 1000 spectra were acquired from all urine samples.

**Table 1.** Demographic table for all cardiac surgery patients.

Characteristics		Age (mean ± SD)61.0 ± 14.8	Gender (M:F)134:66	
Type of cardiac surgery	Coronary artery bypass graft	78 (37.5%)	61:17	
	Heart valve surgery	76 (37%)	41:35	
	Aorta surgery	34 (16.5%)	26:8	
	CABG combined with valve surgery	12 (5.5%)	6:6	
AKI patient’s stages	AKI1	AKI2	AKI3	Total
	42 (52.5%)	19 (23.7%)	19 (23.7%)	80 (54:26)
Gender (M:F)	30:12	14:5	10:9	
Non-AKI patient				120 (80:40)

### 2.2. Data acquisition through Raman spectroscopy

Prior to analysis, samples were removed from the refrigerator and allowed to thaw at room temperature for approximately one hour. Subsequently, 30 µL of each sample was pipetted onto aluminum foil. The Raman spectra of the urine samples were recorded using the Micro Raman Identify system (ProTrusTech, Tainan, Taiwan). An excitation wavelength of 532 nm was used, with a detection wavenumber range of 500–1800 cm^−c^, and each spectrum was scanned for a duration of 15 s. Each spectrum’s background noise was removed, and data redundancy was minimized by applying normalization using MATLAB (R2018a, MathWorks, Natick, MA).

### 2.3. Data analysis

The spectral preprocessed data contained a set of 965 intensity variables from 500 cm^−1^ to 1800 cm^−1^. The classification was performed using SVM. To improve the diagnostic capabilities of the SVM classifier, dimensionality reduction for the urine Raman spectral data was required. PLS was utilized to extract features from the pre-processed Raman spectra of the urine samples. The PLS model was used to predict the concentrations of various biomolecules like proteins, creatine, urea, and uric acid, among others, based on their respective Raman spectra. In this analysis, the latent variables (LVs) are arranged to optimize the distinction between groups. Consequently, the LVs focus on identifying diagnostically relevant changes rather than major variations within the dataset [23]. SVM has proven to be a powerful method for conducting nonlinear classification, multivariate function prediction, and nonlinear regression. By effectively addressing overfitting and underfitting concerns, it produces more reliable and persuasive results [27]. To assess the classification results, the classifier model was fine-tuned using a training dataset, and its performance was evaluated using a test dataset. The first 10 components in the PLS model exhibited a strong correlation coefficient of 0.84 between the *X* and *Y* scores. The output of the PLS model was input into the SVM classifier, which generally divided patients into non-AKI or AKI groups. For each sample measurement, five spectra were gathered from the entire sample area, and the mean of these spectra was utilized in subsequent data analysis. A receiver operating characteristic (ROC) curve was employed to depict the classification of non-AKI and AKI patients, as well as stages with a bias toward non-AKI classification by plotting sensitivity (true positive rate) against 1 −nspecificity (false positive rate).

## 3. Results and discussion

We examined 200 urine samples using RS, of which 140 were utilized to create the AKI detection algorithm or classification model in the training dataset. The other 60 samples were employed for model validation in the testing dataset. The samples were composed of urine from 120 non-AKI patients and 80 AKI patients. Within the AKI patient group, 42 were in stage one, 19 in stage two, and 19 in stage three. Spectra from each stage were individually compared to the non-AKI group to examine the differences in spectral patterns and identify specific variations that could help distinguish between the stages and the non-AKI cases.

### 3.1. Spectral analysis

#### 3.1.1. AKI vs. non-AKI

**Figure 1.**
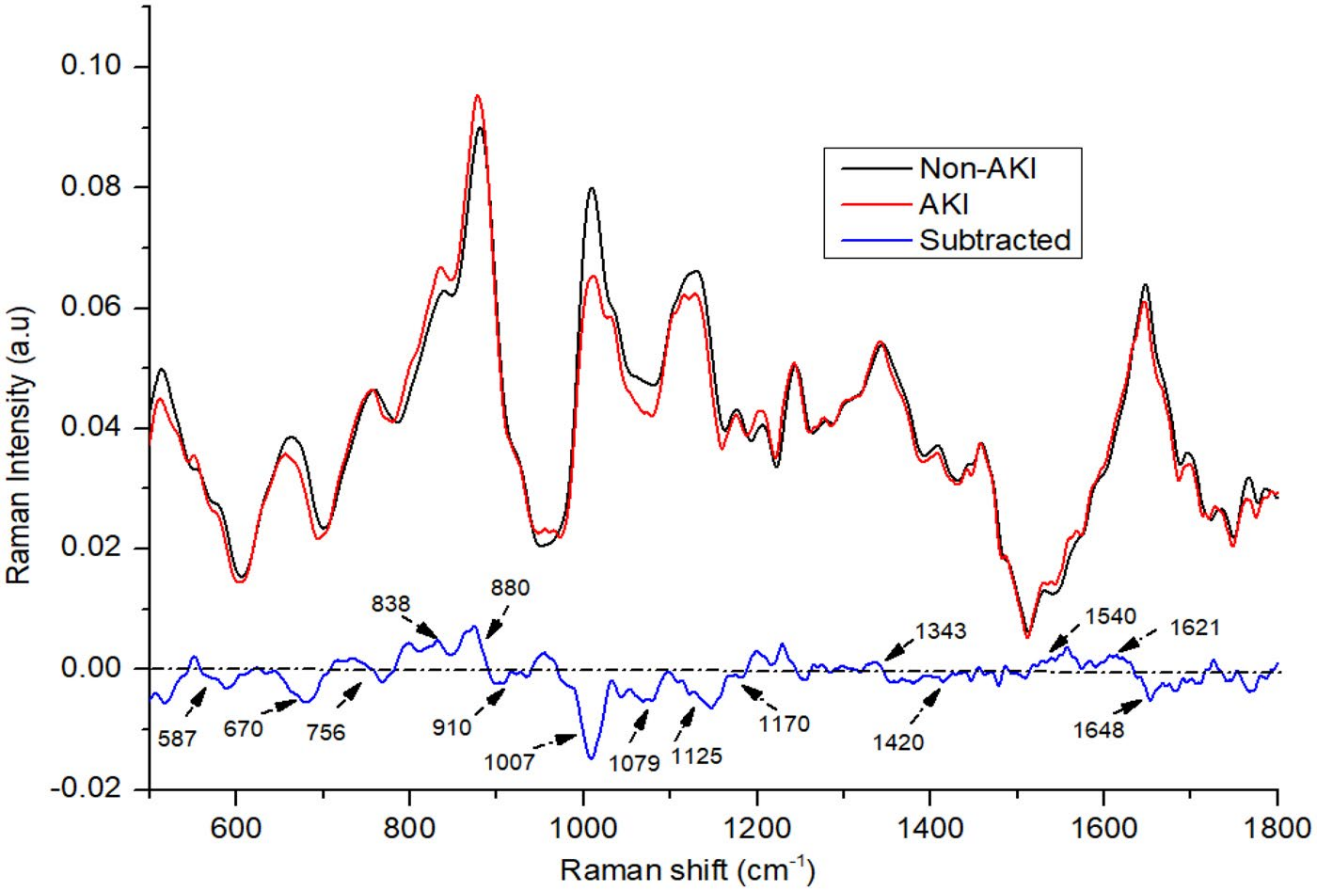
The mean Raman spectra of urine of acute kidney injury (AKI), and non-AKI group.

Figure 1 depicts the averaged, baselined, and vector-normalized urine spectra for both AKI and non-AKI samples. The difference between the Raman spectra of the non-AKI and AKI groups was determined by subtracting the non-AKI group spectra from that of the AKI group. In our investigation, we found that the average spectra for the two groups, AKI and non-AKI, exhibited considerable similarity, with the primary constituents consisting of urea, creatinine, uric acid, porphyrin, nitrogenous compounds, tryptophan, and uric acid. The AKI spectra exhibited more pronounced positive or negative peaks for nitrogenous compounds (880 and 1079 cm^−1^), tryptophan (838 cm^−1^), neopterin (1540 cm^−1^), and porphyrin (1621 cm^−1^) compared to the non-AKI spectra. In contrast, the non-AKI urine samples displayed noticeable peaks for urea (587, 1007, and 1170 cm^−1^), creatinine (670, 910, and 1420 cm^−1^), and uric acid (1648 cm^−1^) [28–30]. The nitrogenous compounds, predominantly manifested as ethanolamine bands at 880 cm^−1^, likely resulted from amino acid metabolism in the urea cycle. Higher concentrations of these compounds were detected in the urine of patients with chronic kidney disease [31]. In cases of kidney disease, the metabolic capacity of the kidney is diminished, leading to decreased signal intensities for substances such as urea and creatinine, as shown in Figure 1. Urea and creatinine are crucial urine components that offer valuable insights into kidney health and can aid in the identification of early-stage renal disorders [32,33]. Tryptophan, an amino acid, exhibited higher levels in AKI patients compared to the non-AKI group. A small peak, appearing at 1456 cm^−1^, was observed in the urine samples from both groups, this peak can be attributed to hydroxybutyrate, as depicted in Figure 1. A subtle peak corresponding to the vibration of neopterin at 1540 cm^−1^ was observed in both AKI and non-AKI subjects; however, the peak intensity was higher in AKI patients compared to the non-AKI individuals. Both groups demonstrated the presence of uric acid, evidenced by the spectral peak at 1648 cm^−1^ [22]. However, slight variations in intensity were observed, with the non-AKI group presenting a higher intensity for uric acid. Additionally, two small and broad creatinine peaks at 670 cm^−1^ were observed, exhibiting higher intensity in the control subjects. A peak at 1125 cm^−1^ exhibited higher intensity in non-AKI urine samples, which may be attributed to the presence of indoxyl sulfate. On the other hand, peaks corresponding to hydroxybutyrate (1456 and 1343 cm^−1^) exhibited greater intensity in the urine samples of AKI subjects compared to those from patients with non-AKI. The spectral peak observed at 756 cm^−1^ is also associated with tryptophan, an amino acid prevalent in proteins. Interestingly, this peak exhibited a marginally higher intensity in the non-AKI group compared to the AKI group.

#### 3.1.2. AKI 1 vs. non-AKI

**Figure 2.**
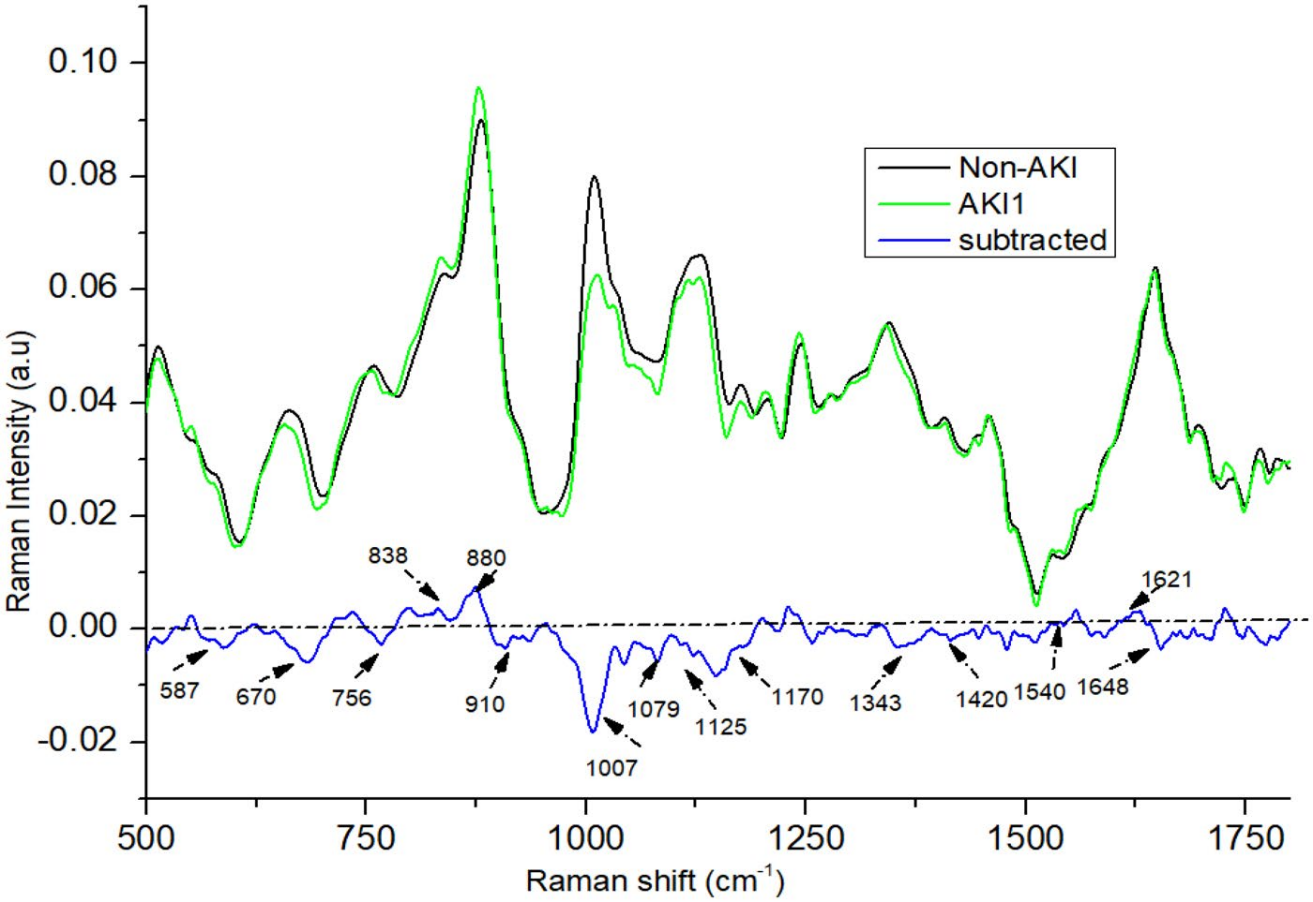
The mean Raman spectra of urine of acute kidney injury (AKI) stage 1, and non-AKI group.

The Raman spectra for stage 1 (as shown in Figure 2) AKI show a remarkable resemblance to the mean AKI Raman spectra, with subtle differences noted particularly around the peaks at 1420, 1170, 1343, 1540, and 1648 cm^−1^. Notably, the intensity of the peaks at 1420 and 1648 cm^−1^ in the stage 1 AKI spectra is higher than in the mean AKI spectra. In contrast, the peaks at 1540, 1170, and 1343 cm^−1^ manifest lower intensities in stage 1 AKI compared to the mean AKI spectra. These slight yet significant spectral variations offer adequate differentiation, enabling effective segregation of the stages.

#### 3.1.3. AKI 2 vs. non-AKI

**Figure 3.**
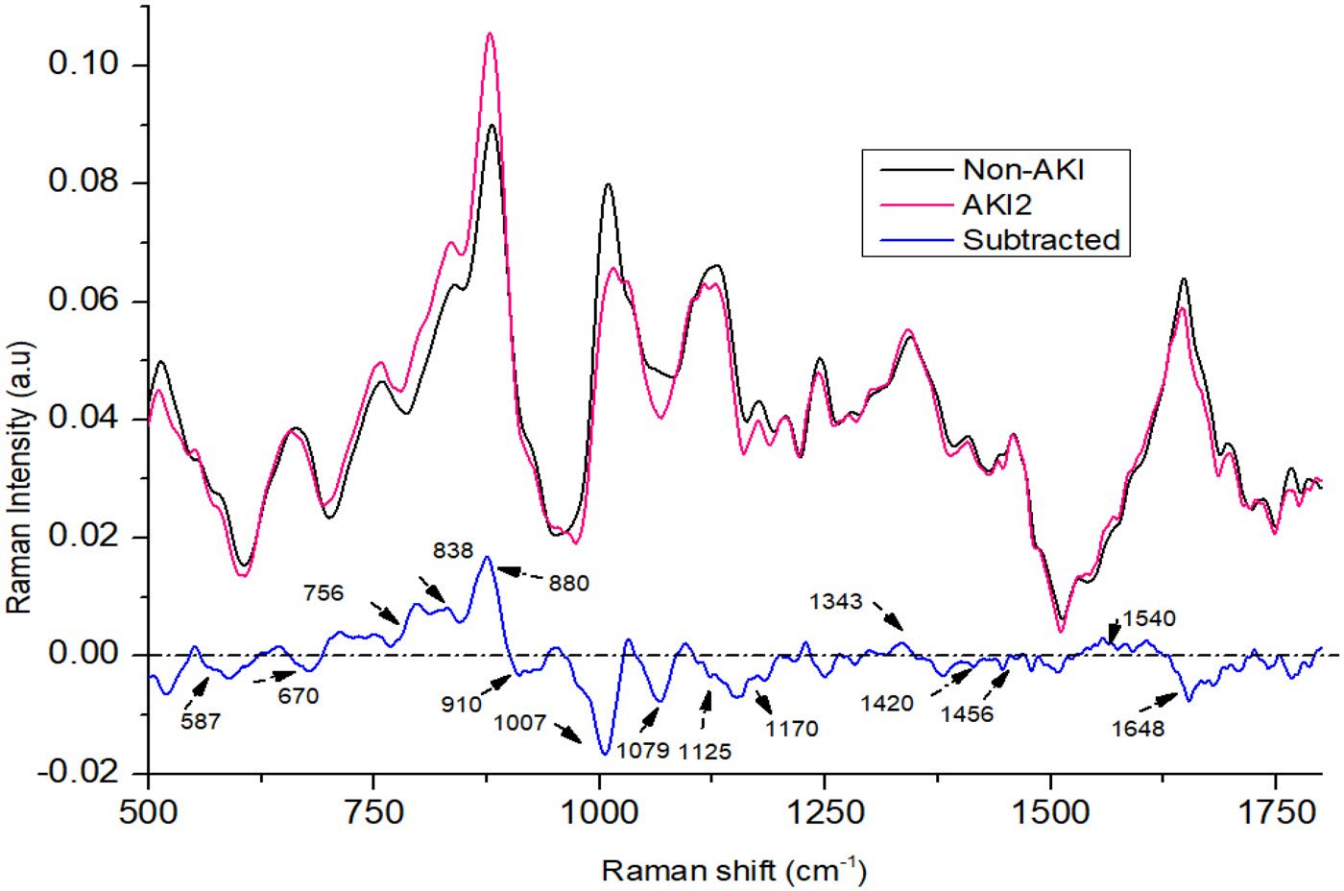
The mean Raman spectra of urine of acute kidney injury (AKI) stage 2, and non-AKI group.

In the case of AKI stage 2, the spectral peaks at 756, 838, 880, 1079, 1343, and 1540 cm^−1^ showed higher intensities compared to the non-AKI group as shown in Figure 3. Conversely, the peaks at 670, 910, 1007, 1125, 1170, 1420, and 1648 cm^−1^ were more pronounced in the non-AKI group. At 838 cm^−1^ and 880 cm^−1^ and 756 cm^−1^, the intensity is highest in AKI stage 2 compared to AKI1 and AKI3. The peak at 1343 cm^−1^ is also more intense in AKI stage 2 than AKI stage 1. The peak at 1648 cm^−1^ is less intense in AKI stage 2 compared to AKI1 and AKI3.

#### 3.1.4. AKI 3 vs. non-AKI

**Figure 4.**
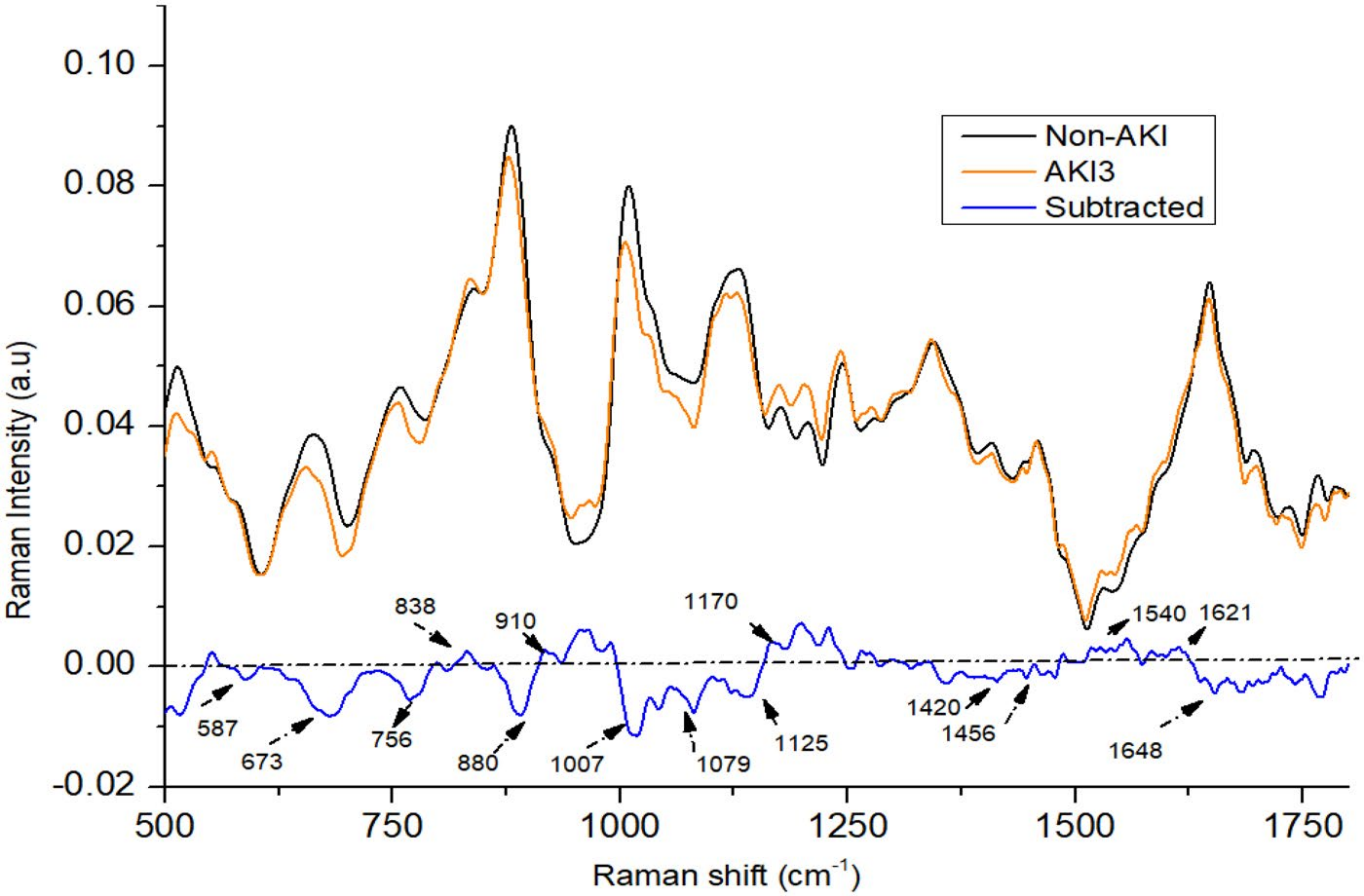
The mean Raman spectra of urine of acute kidney injury (AKI) stage 3, and non-AKI group.

In the AKI stage 3 patients, the intensity at peaks 587, 673, 756, 880, 1007, 1420, and 1648 cm^−1^ was lower in comparison to non-AKI patients (as shown in Figure 4). Conversely, the intensity at peaks 838, 910, 1079, 1170, 1540, and 1621 cm^−1^ was higher in AKI stage 3 patients compared to the non-AKI group. In AKI3 patients, it was found that peak at 880 cm^−1^ was weaken than non-AKI patients, it may be due to the elevated levels of nitrogenous compounds in urine samples which could result from consumption of certain drugs. The spectral peak at 1648 cm^−1^ in AKI stage 3 patients exhibits a higher intensity compared to those in AKI stage 2 patients, yet it displays lower intensity when compared to AKI stage 1 patients. Meanwhile, the spectral peaks at 673, 1343, and 1420 cm^−1^ appear less intense in AKI stage 3 patients compared to those in AKI stage 2. Conversely, the intensities of the spectral peaks at 1170, 1540, and 1648 cm^−1^ are higher in AKI stage 3 patients compared to AKI stage 2 patients. Certain medications can have an impact on kidney function and subsequently affect the concentrations of creatinine, urea, and uric acid or other metabolites present in urine and blood [34,35]. This could possibly account for the observed variations in certain chemical constituents within urine samples from patients in AKI stages 2 and 3, thereby influencing the results of the study.

### 3.2. Fine-tuning and performance evaluation of PLS components

**Figure 5.**
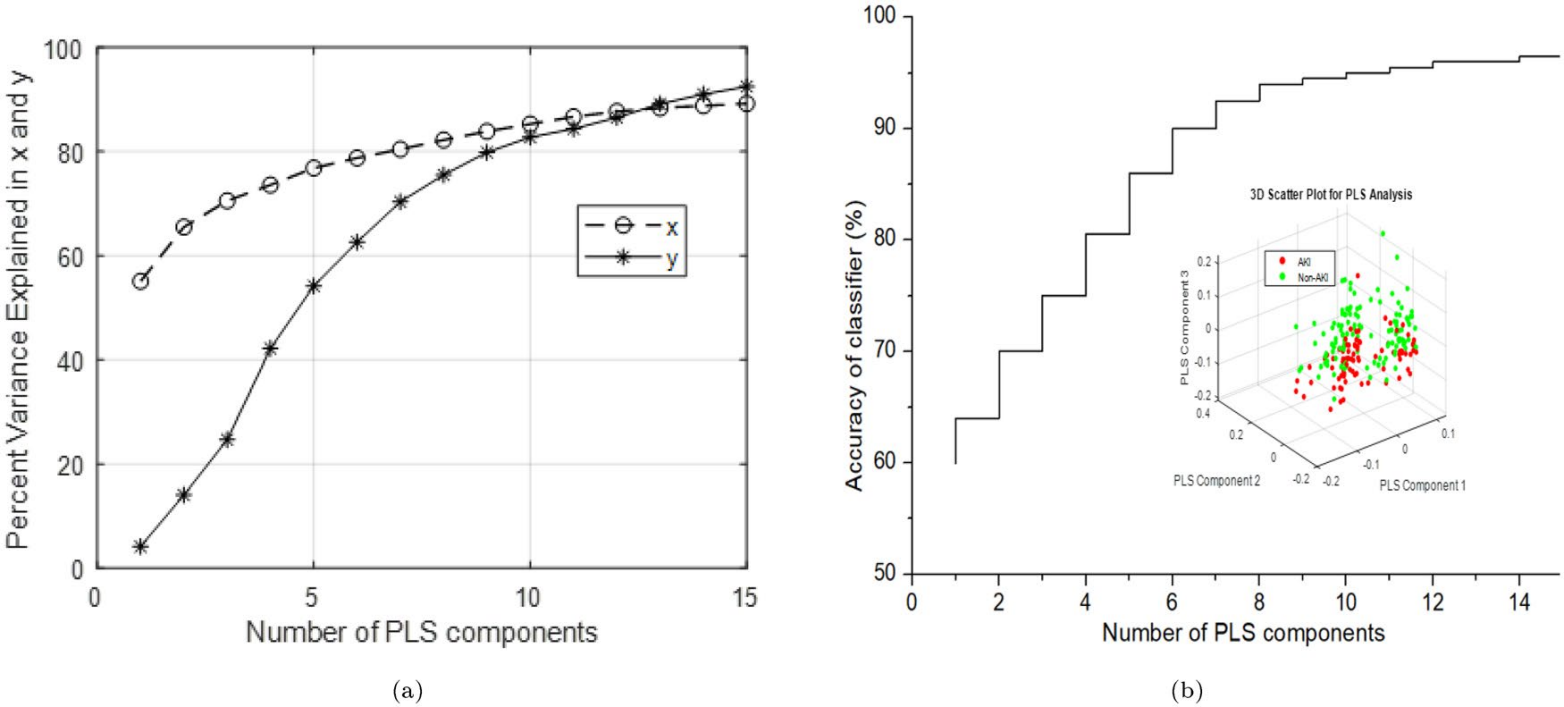
Screen plot of the cumulative percentages of the first 15 PLS components (a) with the percentage variance of *x* (predictors) and *y* (response variables) (b) with the accuracy curve of PLS-SVM algorithm, the inset shows the PLS-scatter plot.

Upon utilizing 10 PLS components, the graph demonstrates that the explained variance for both predictors and the response variable attains a saturation point, exhibiting minimal increase beyond this point, as shown in Figure 5(a). In the PLS model, the first 10 components exhibit a robust correlation coefficient of 0.99 between the *X* and *Y* scores. For 10 PLS components, the *R*-squared value and mean squared error are calculated as 0.98 and 0.0325, respectively. *K*-fold cross-validation method was also used for verifying the number of optimized PLS components, which typically offers an impartial estimate of the model’s true accuracy. A larger validation value is advised, as it strikes an optimal balance between bias and variance [36]. A PLS-SVM model was evaluated using a test dataset with PLS components ranging from 1 to 15 vs. accuracy as illustrated in Figure 5(b). The saturation point observed for accuracy as a function of the number of PLS components supports the 10 PLS components determined via the *K*-fold cross-validation method. These significant PLS components were subsequently employed as input for the SVM analysis.

### 3.3. Training and validation of model

In our methodology, we partition the dataset into two segments: 70% for training the model and 30% for evaluating its performance. This strategy aids in assessing the model’s generalization capabilities on unseen data, minimizing the likelihood of over-fitting. To split the dataset, we randomly shuffle the data and designate 70% of the samples for training, with the remaining 30% allocated for testing. To enhance the dependability of the performance assessment, we execute the splitting process multiple times, each instance generating a new training and testing set. This technique (repeated random sampling) was referred for assessing the performance and stability of a model [37]. By computing the mean of performance metrics (such as sensitivity and specificity) across multiple iterations, we can derive a more precise and reliable assessment of the model’s performance.

### 3.4. Evaluation of model using testing set

*K*-fold cross-validation was employed to enhance the reliability and generalizability of the SVM classifier’s performance during model evaluation. The dataset is split into *k* equal-sized folds in *k*-fold cross-validation. The model is trained on *k* −f1 folds and tested on the remaining fold, with the process repeated *k* times, ensuring each fold serves as the test set once. Average performance metrics are calculated by aggregating the results across all *k* iterations. Tables 2 and 3 present the comprehensive performance metrics for classifying AKI and non-AKI cases, encompassing all stages. Balanced accuracy is preferred over accuracy in imbalanced datasets, as it fairly considers classifier performance on both majority and minority classes, avoiding misleading results from unequal class distribution [38].

**Table 2.** Classification parameters of AKI and non-AKI group using PLS-SVM classifier: SEN (sensitivity), SPE (specificity), AC (accuracy), *F*1 (*F*1-score), BAC (balanced accuracy), and MCC (Matthew’s correlation coefficient).

PLS-SVM	SEN (%)	SPE (%)	AC (%)	PRE (%)	BAC (%)	*F*1-score (%)	MCC
Parameters	91.7	94.4	93.3	91.7	93	91.7	0.861

**Table 3.** Stage bias classification parameters of AKI and non-AKI group using PLS-SVM classifier: SEN (sensitivity), SPE (specificity), AC (accuracy), precision (PRE), *F*1 (*F*1-score), BAC (balanced accuracy), and MCC (Matthew’s correlation coefficient).

Parameters	SEN (%)	SPE (%)	AC (%)	PRE (%)	BAC (%)	*F*1-score (%)	MCC
AKI1 vs. non-AKI	90	97.4	95.9	90	93.7	90	0.874
AKI2 vs. non-AKI	80	100	97.6	100	90	88.9	0.883
AKI3 vs. non-AKI	83.3	100	97.6	100	91.7	90.9	0.9

**Figure 6.**
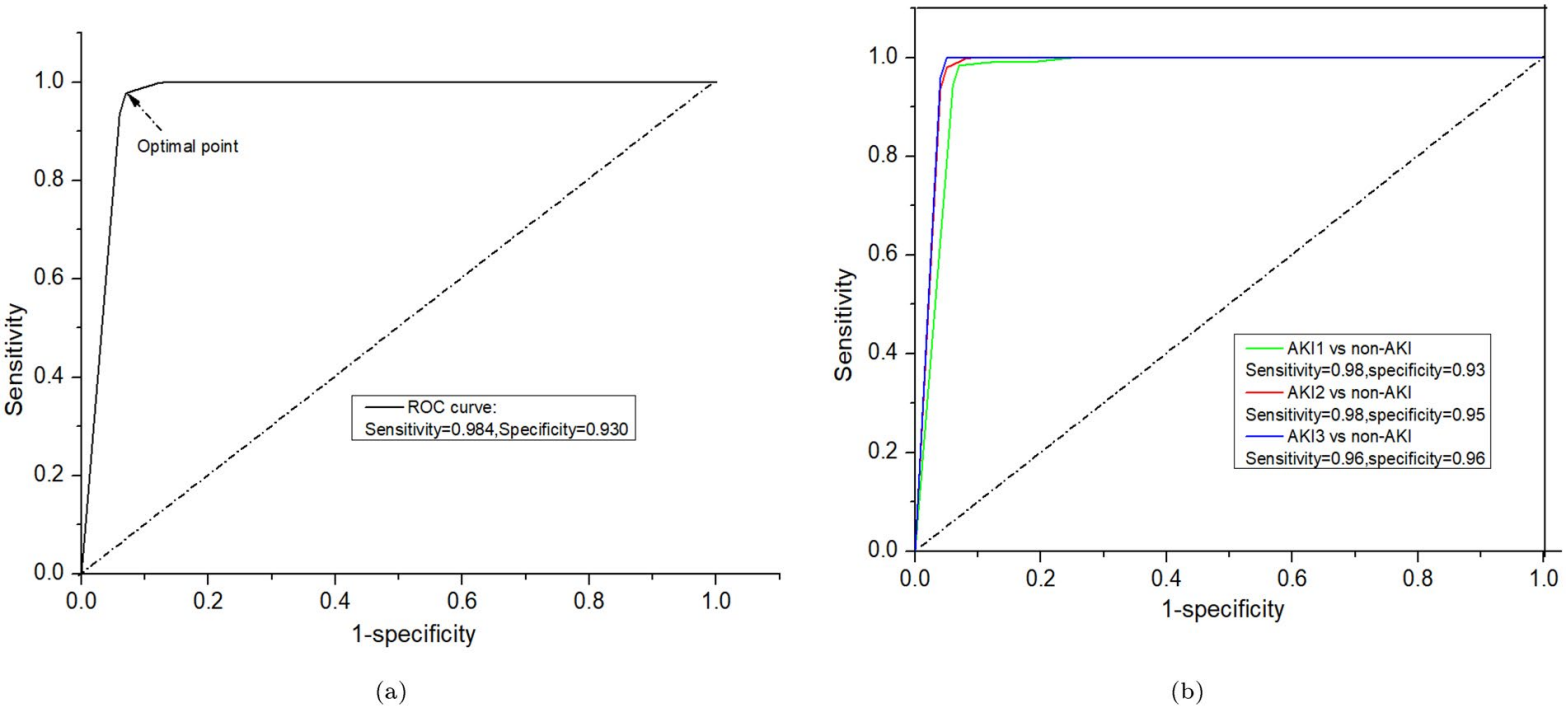
ROC curves for classification results using PLS-SVM: (a) AKI vs. non-AKI factors and (b) non-AKI vs. individual AKI stages.

Utilizing *k*-fold cross-validation offers a more robust performance estimate for the SVM classifier, reducing over-fitting risks and providing better insight into the model’s expected performance on unseen data. The PLS-SVM model was evaluated using various metrics, including sensitivity, specificity, accuracy, *F*1-score, balanced accuracy, Matthew’s correlation coefficient, and area under the curve (AUC). This model achieved a highest balanced accuracy of 93%, a sensitivity of 91.7%, and a specificity of 94.4% for classification (Table 2). The ROC curves for the PLS-SVM model are displayed in Figure 6, indicating that the maximum value of AUC exceeded 95%. This high AUC value highlights the model’s ability to effectively distinguish AKI cases from non-AKI, indicating robust classification capabilities and potential practical applications in the given context. The ROC curve was generated using both training and testing datasets. The testing dataset provided an accuracy of 97.5% with 98.9% AUC, as exhibited in Figure 6(a). Further, non-AKI patients were successfully distinguished from patients with AKI stages 1, 2, and 3, highlighting the model’s ability to differentiate between healthy individuals and those with various stages of AKI. The balanced accuracy is 93.7% in the AKI stage 1 and non-AKI group, 90% in the AKI stage 2 and non-AKI group, and 91.7% in the AKI stage 3 and non-AKI group, with all values around 90%. The sensitivity and specificity are 90%, 97.4%, and 80% and 100%, 83.3%, and 100%, respectively, as shown in Table 3. These results demonstrate that the diagnostic model is stable and can effectively identify urine samples from patients with various stages of AKI, showcasing its potential for use in clinical settings. Afterwards, evaluation of the performance of model ROC curve has been drawn for each class of AKI and non-AKI as shown in Figure 6(b), which is also a good agreement of above results.

Our study showcases the effectiveness of RS as a potent tool for diagnosing AKI through urine sample analysis. Urine, a noninvasively collectible kidney byproduct, is a promising medium for renal biomarker studies. AKI severity, as per the Kidney Disease: Improving Global Outcomes (KDIGO) guidelines, is categorized into three distinct stages (AKI stage 1, 2, and 3). This categorization is primarily based on specific criteria such as the levels of serum creatinine, decreased urine output, and the requirement for renal replacement therapy. In this study, we also successfully distinguished each of these three AKI stages from non-AKI patients. The application of RS in identifying and tracking the excretion rate of chemical constituents in urine such as nitrogenous compounds, urea, creatinine, and uric acid, showcasing its potential for breakthroughs in this research field [39]. Creatinine is a significant component in various biological processes and its measurement is a widely used method for assessing kidney function [32]. Urinary creatinine concentration measurement is utilized to evaluate kidney function adequacy and the severity of kidney damage, as well as to monitor the progression of kidney disease. Creatinine clearance, which represents the kidneys’ ability to remove creatinine from the blood, is also employed as an indicator of renal function [40]. Urea production primarily takes place in the liver as a result of protein metabolism. It is then transported in the blood to the kidneys, where it is filtered from the plasma by the glomerulus. Measuring urea concentration is crucial for evaluating renal function (diagnosing kidney disease), confirming the effectiveness of dialysis, determining nitrogen balance, and assessing hydration status [41]. Uric acid is a waste product excreted by the kidneys. During the early stages of AKI, uric acid levels in the urine might decrease, as the kidneys’ ability to expel this waste product is compromised. In our study, we sourced all urine samples from heart surgery patients, aiming to achieve enhanced sensitivity and specificity through the application of the PLS-SVM classification model. AKI patients have a lower amount of urea, creatinine, and uric acid in comparison to non-AKI patients. We observed a decrease in the uric acid assignment in the later stages of AKI (stages 2 and 3) compared to the early stage (AKI 1). The presence of urea, identified at 1170 cm^–1^, was diminished in AKI 1 and AKI 2 but elevated in AKI 3. However, this observation is not significant as medications could cause variations in urea levels. Furthermore, the peaks associated with creatinine (673 and 1420 cm^–1^) were noticeably weaker in AKI 3 patients compared to those in stages 1 and 2. This indicates a corresponding decrease in creatinine levels as the condition progresses to more advanced stages.

A PLS-SVM model was used to analyze the statistical efficiency of this tool. PLS is a dimensionality reduction technique used to extract the most relevant features from the data, while SVM is a supervised machine learning algorithm used for classification or regression tasks. The PLS-SVM model is preferable to spectral data and is the most effective multivariate statistical technique [42]. In current study, a ROC curve was used to evaluate the clinical potential of the PLS-SVM model, with the AUC offering an estimation of sensitivity and specificity at various significance levels. These results suggest that the classification model has great potential for accurate and reliable diagnosis in clinical settings. However, further validation with larger datasets is recommended to ensure the robustness and applicability of the model in real-world scenarios. Utilizing RS, we can swiftly analyze the biomolecules present in urine, a process that could significantly mitigate the incidence of late detection. The identification of these unique biomarkers is central to our study, as we strive to facilitate early detection and timely intervention, thereby enhancing the clinical outcomes for patients. One study offers significant insights into the role of NAD + metabolism in AKI, particularly for patients undergoing cardiac surgery [43]. It can be noticed that renal NAD + level decrease in AKI, and restoring NAD + levels via NAD + precursor supplementation may protect against AKI. One source of tissue NAD + is tryptophan metabolism. This study emphasizes the urinary quinolinate/tryptophan ratio (uQ/T) as a potential biomarker for AKI. Quinolinate, a key intermediate in the *de novo* NAD + biosynthetic pathway from tryptophan, is converted to NAD + by the enzyme quinolinate phosphoribosyltransferase (QPRT). However, there is a block in NAD + synthesis, indicated by the elevated uQ/T ratio, likely due to reduced QPRT activity. This blockage, despite the abundance of tryptophan, could be a critical factor in AKI pathogenesis. The study also shows that NAD + augmentation through nicotinamide (NAM) administration is beneficial, increasing circulating NAD + metabolites and reducing AKI incidence. This supports the potential of NAD + augmentation strategies as a therapeutic approach for high-risk patients, including those undergoing cardiac surgery.

Furthermore, our primary objective is to establish correlations between surgical types and the diabetes status of patients, concentrating on specific chemical constituents that show potential for diagnosing AKI and non-AKI conditions. To enhance clarity, in the future, we plan to incorporate a study offering detailed information on the surgery types and preexisting disease statuses of the patients, and how the average Raman spectra for each class can be influenced by these factors.

Early intervention for AKI is associated with improved prognosis in renal diseases. The KDIGO guidelines for AKI recommend initiating several supportive measures in patients at high risk [44]. These measures include close hemodynamic monitoring using devices like a pulse index continuous cardiac output (PICCO) catheter or FloTrac system to optimize volume status and hemodynamic parameters, close monitoring of serum creatinine, urine output, and fluid balance, avoidance of hyperglycemia, considering alternatives to radiocontrast procedures, and avoiding nephrotoxic agents. Identifying urine biomarkers for prompt diagnosis could allow for earlier identification of high-risk patients and enhance the effectiveness of timely intervention strategies, thereby reducing the frequency and severity of AKI, as demonstrated in some studies [45,46]. Our study aims to address this gap by investigating the potential of RS for detecting early signs of AKI and guiding timely interventions. Our findings revealed distinct metabolic profiles for each AKI stage: stage 1 exhibited high uric acid and low creatinine levels; stage 2 showed increased tryptophan and nitrogenous compounds but lower uric acid; and stage 3 displayed high neopterin and low creatinine levels. Following cardiac surgery, our Raman analysis enables the immediate identification of abundant or deficient compounds in patients, allowing clinicians to initiate early treatment accordingly. We recognize the importance of further research and clinical studies to translate these findings into actionable interventions. Collaborative efforts with healthcare providers and researchers are essential to explore the feasibility and effectiveness of implementing early interventions, such as utilizing RS for early AKI diagnosis, in clinical practice. An episode of AKI is linked to both short-term complications like fluid overload, electrolyte imbalances, immune dysfunction, and bleeding issues, as well as long-term adverse effects on survival. Mild AKI, classified as stage 1 by the KDIGO guidelines, is linked to decreased survival rates, with effects persisting for a decade or longer [47].

## 4. Conclusions

The distinct spectral variations observed in the urine samples of AKI patients compared to non-AKI patients could be ascribed to changes in the composition and structure of urine metabolites, such as urea, creatinine, uric acid, nitrogenous compounds, and tryptophan. By applying PLS-SVM analysis to the spectral data, we achieved an overall accuracy of approximately 93%. Furthermore, the model accurately differentiated between non-AKI patients and those with AKI stages 1, 2, and 3, with respective accuracies of 94%, 90%, and 92%. In the future, this study could potentially catalyze transformative advancements in the detection and monitoring of AKI, thereby augmenting both the efficiency and effectiveness in clinical practice.

## Supplementary Material

The_clean_editable_revised_paper.7z

## Data Availability

Data are available from the corresponding author upon reasonable request.
